# The Bacterial Community in the Western Region of North Pacific Intermediate Water

**DOI:** 10.1128/MRA.00757-19

**Published:** 2019-08-22

**Authors:** Maki Teramoto, Ayumi Komatsu, Kouhei Ohnishi

**Affiliations:** a Oceanography Section, Kochi University, Nankoku, Kochi, Japan; b Research Institute of Molecular Genetics, Kochi University, Nankoku, Kochi, Japan; Georgia Institute of Technology

## Abstract

The bacterial composition in North Pacific Intermediate Water (NPIW) was investigated in three different years and compared with that in other seawaters around Japan. The results indicated that bacterial composition was surprisingly stable at the same point in a mesopelagic water mass throughout the years and supported previous physicochemical observations that NPIW is distributed to Kumejima, Japan.

## ANNOUNCEMENT

Bacterial distribution is related to hydrography and water masses in deep-sea environments (1, 2). However, long-term changes in bacterial distribution remain largely unknown in mesopelagic water masses (200 to 1,000 m depth). Therefore, in this study, bacterial composition was investigated in a mesopelagic water mass, North Pacific Intermediate Water (NPIW), at Muroto in Japan (3) (http://www.jamstec.go.jp/J-ARGO/index_e.html) in three different years. For comparison, other mesopelagic water masses, the Rausu water mass and Japan Sea Proper Water (3) (http://www.jamstec.go.jp/J-ARGO/index_e.html), as well as surface seawaters around Japan were also investigated.

The analyzed seawaters are summarized in Table 1. Mesopelagic waters were sampled by local facilities such as the Kochi Prefectural Deep Seawater Laboratory. Microorganisms in seawater (12 to 30 liters for mesopelagic water and 2 to 6 liters for surface seawater) were collected on a filter with 0.22-μm pores (Express Plus; Millipore) immediately after fresh seawater was obtained or stored at 4°C overnight. The bacteria on the filters were immediately stored at −80°C or dried at 55°C and kept dry before storage at −80°C until DNA extraction.

**TABLE 1:** Seawater samples analyzed in this study and the number of 16S rRNA gene fragment sequences analyzed and species-level OTUs found for the sequences from each seawater sample

Sample type by location (depth [m])	Coordinates	Mesopelagic water mass^*a*^	Time (mo/yr)	Temp (°C)^*b*^	No. of 16S rRNA gene fragment sequences^*c*^	No. of OTUs^*d*^	DDBJ accession no.
Pacific							
Muroto, Kochi							
Mesopelagic (320)	33°18′N, 134°14′E	NPIW	7/2011	ND	7,746	2,331	DRA001052
Mesopelagic (320)	33°18′N, 134°14′E	NPIW	1/2012	ND	1,321	650	DRA005075
Mesopelagic (320)	33°18′N, 134°14′E	NPIW	11/2013	ND	1,404	543	DRA005076
Surface (0.5)	33°18′N, 134°11′E		7/2011	25.7	7,513	2,853	DRA001054
Surface (0.5)	33°18′N, 134°11′E		11/2011	21.3	7,119	2,375	DRA001053
Okinawa							
Mesopelagic at Kumejima (612)	26°23′N, 126°48′E	“NPIW”	11/2013	ND	1,629	586	DRA005079
Surface at Okinawa Main Island (0)	26°23′N, 127°59′E		10/2013	25.2	1,675	550	DRA005081
Surface at Ishigaki Island (0)	24°27′N, 124°9′E		4/2013	22.3	867	337	DRA005080
Suruga, Shizuoka							
Mesopelagic (397)	34°51′N, 138°21′E	“NPIW”	11/2011	ND	7,769	2,811	DRA001055
Surface (0)	34°38′N, 138°24′E		11/2011	23.5	9,048	1,723	DRA001056
Rausu, Hokkaido							
Mesopelagic (350)	44°37′N, 145°14′E	“Rausu”	11/2013	ND	1,329	533	DRA005078
Japan Sea							
Noto, Ishikawa							
Mesopelagic (320)	37°17′N, 137°16′E	“JSPW”	11/2011	ND	8,445	2,288	DRA001057
Surface (0)	37°18′N, 137°14′E		11/2011	20.5	8,008	1,442	DRA001058
Iwanai, Hokkaido							
Mesopelagic (300)	43°00′N, 140°25′E	“JSPW”	11/2013	ND	1,799	796	DRA005077
Total					65,672	15,520	

a The original water masses were deduced from the description by Taniguchi (3) and from temperature-salinity data, close to the sampling locations, from Argo JAMSTEC (http://www.jamstec.go.jp/J-ARGO/index_e.html). NPIW, North Pacific intermediate water; “NPIW”, probably NPIW; “Rausu”, probably Rausu water mass; “JSPW”, probably Japan Sea Proper Water.

b ND, no data available.

c The gene fragment sequences ranged in size from 230 to 562 bp.

d OTUs were detected using QIIME version 1.9 for all seawater samples (65,672 sequences) and not for each seawater sample.

DNA extraction, PCR amplification of the V1 to V3 region of the 16S rRNA bacterial gene, emulsion-based clonal amplification using a Lib-A kit (Roche), and sequencing on the GS Junior 454 system (Roche) were conducted as described previously (4). Tag and primer regions used for PCR were removed from the sequences by QIIME (http://qiime.org/) (5) and manually in Se-Al after alignment in ClustalX (version 2.1) (6). Chimeric sequences were checked and removed by either Bellerophon (7) and Mallard (8) or DECIPHER (9) and USEARCH 6.0 (http://fungene.cme.msu.edu/FunGenePipeline/chimera_check/form.spr) (10, 11) programs. Furthermore, sequences considered nonbacterial by the Ribosomal Database Project Classifier with a confidence threshold of 80% (12, 13) were removed. Then, the remaining 65,672 sequences were analyzed in this study.

QIIME version 1.9 was used for clustering the 65,672 sequences into operational taxonomic units (OTUs; maximum distance of 0.03, grouping at the species level) using uclust (10), selecting representative sequences from each OTU (10), and assigning their taxonomy (14, 15) against the BLAST database (16). Based on the percent OTU composition profiles of waters, a nonmetric multidimensional scaling (NMDS) plot based on Bray-Curtis similarity was constructed using the PAST software (version 2.15) (17). Default parameters were used for all software programs, unless otherwise stated.

NMDS and abundant OTU results showed that bacterial species composition was quite stable in NPIW at Muroto through seasons and years, indicating that the composition was stable at the same point in a mesopelagic water mass through the years. The results also showed that the composition at Kumejima was closely related to that in NPIW at Muroto, supporting previous physicochemical data that NPIW is distributed to Kumejima.

Characteristically abundant OTUs in (probable) NPIW belonged to *Pelagibacteraceae*, *Piscirickettsiaceae*, SAR406, and SAR202. SUP05 bacteria (18–21) seemed to be highly abundant in probable NPIW at Suruga.

### Data availability.

The sequences used were deposited in the DDBJ Sequence Read Archive under the accession numbers listed in Table 1.
